# Large-Scale Monitoring of Plants through Environmental DNA Metabarcoding of Soil: Recovery, Resolution, and Annotation of Four DNA Markers

**DOI:** 10.1371/journal.pone.0157505

**Published:** 2016-06-16

**Authors:** Nicole A. Fahner, Shadi Shokralla, Donald J. Baird, Mehrdad Hajibabaei

**Affiliations:** 1 Department of Integrative Biology and Biodiversity Institute of Ontario, University of Guelph, Guelph, Ontario, Canada; 2 Environment Canada at Canadian Rivers Institute and Department of Biology, University of New Brunswick, Fredericton, New Brunswick, Canada; University of Saskatchewan, CANADA

## Abstract

In a rapidly changing world we need methods to efficiently assess biodiversity in order to monitor ecosystem trends. Ecological monitoring often uses plant community composition to infer quality of sites but conventional aboveground surveys only capture a snapshot of the actively growing plant diversity. Environmental DNA (eDNA) extracted from soil samples, however, can include taxa represented by both active and dormant tissues, seeds, pollen, and detritus. Analysis of this eDNA through DNA metabarcoding provides a more comprehensive view of plant diversity at a site from a single assessment but it is not clear which DNA markers are best used to capture this diversity. Sequence recovery, annotation, and sequence resolution among taxa were evaluated for four established DNA markers (*mat*K, *rbc*L, ITS2, and the *trn*L P6 loop) *in silico* using database sequences and *in situ* using high throughput sequencing of 35 soil samples from a remote boreal wetland. Overall, ITS2 and *rbc*L are recommended for DNA metabarcoding of vascular plants from eDNA when not using customized or geographically restricted reference databases. We describe a new framework for evaluating DNA metabarcodes and, contrary to existing assumptions, we found that full length DNA barcode regions could outperform shorter markers for surveying plant diversity from soil samples. By using current DNA barcoding markers *rbc*L and ITS2 for plant metabarcoding, we can take advantage of existing resources such as the growing DNA barcode database. Our work establishes the value of standard DNA barcodes for soil plant eDNA analysis in ecological investigations and biomonitoring programs and supports the collaborative development of DNA barcoding and metabarcoding.

## Introduction

Monitoring changes in biodiversity at a site over time–“biomonitoring”–is key for understanding ecosystem status [1,2]. Plant communities are regularly assessed in biomonitoring programs, however, aboveground morphological surveys only capture a snapshot of existing plant growth and may fail to observe any species missing diagnostic characters such as flowers [3] as well as ephemeral, cryptic or dormant plants [4]. Molecular methods such as DNA barcoding—specimen identification by sequencing a standardized genomic region and comparing it against a reference database—are increasingly being used [5] but still require collection and separation of individual specimens [6], and are unsuitable for surveys of belowground plant diversity [7].

Marker gene sequences from environment samples have been used in metagenomic [4,5] and ancient DNA analysis [8]. More recently, in line with advancements of high throughput sequencing, DNA metabarcoding is formally proposed to increase the efficiency and scale of ecological assessments [1,2,9–11]. DNA metabarcoding is the simultaneous characterization of whole communities from unsorted bulk samples. For biomonitoring, environmental DNA (eDNA) extracted from samples of soil or water is subjected to high throughput sequencing (HTS) and sequences are compared to reference libraries to identify the biodiversity at a given site. Soil eDNA includes DNA from active and dormant plant tissues, seeds, pollen and plant detritus [4,12], and can potentially reveal a site’s total plant diversity [12]. Not only can plant DNA metabarcoding provide new insights for biomonitoring but it has already led to novel avenues for forensic soil analysis [13] and enriched our understanding of animal diets [14,15].

Plastid genes *rbc*L and *mat*K were previously chosen as the official two-locus plant DNA barcode based on Sanger sequencing of individual specimens [16,17] and follow-up studies showed that taxonomic resolution is improved by adding sequence information from the nuclear ribosomal internal transcribed spacer (ITS) [17–19]. The non-coding plastid *trn*L (UAA) intron P6 loop, however, is currently promoted as the most suitable marker for plant eDNA metabarcoding, mainly due to its short 10–143 bp length [12,20–22]. While this length can be more efficient for analysis of degraded DNA, species resolution is minimal unless specially curated reference databases are used [21].

Unlike standard single-specimen DNA barcoding, environmental samples routinely include mixed templates representing an unknown number of taxa [23] and each DNA marker must independently identify taxa because sequences cannot be combined in a multigene tiered approach (e.g.[24]). Instead, the taxonomic composition observed at a site with eDNA relies on the sequence recovery, sequence resolution among taxa, and annotation of individual markers. In other words: 1. Are sequences of sufficient quality and length recovered for all taxa present at a site? 2. Is there enough molecular divergence at the locus to distinguish taxa from one another? 3. Can complete and correct taxonomy be assigned to sequences using reference databases? Together these factors explain why different DNA markers may report different plant communities for the same sample.

Previous comparisons of DNA markers for metabarcoding were primarily *in silico*, emphasized primer design, and based conclusions on assumptions about length of DNA fragments that can be recovered from soil (i.e. <200 bp) [20,22,25]. Here, we systematically evaluate the suitability of these four established DNA markers (*mat*K, *rbc*L, ITS2, and *trn*L P6 loop) for biodiversity assessment of vascular plants through DNA metabarcoding. First, we conducted *in silico* tests with reference database sequences to evaluate annotation and sequence resolution when taxonomic identities are known. Second, *in situ* tests with 35 soil samples from boreal wetlands were used to compare sequence recovery, annotation, and taxon resolution. Finally, we examined taxonomic breadth and overall complementarity of each locus resulting from cumulative differences in recovery, annotation, and resolution of vascular plant sequences.

## Materials and Methods

### Study Site

Soil samples were collected from four long term study sites in the Ramsar designated Peace-Athabasca Delta (PAD) wetlands of Wood Buffalo National Park, Alberta, Canada through the Biomonitoring 2.0 pilot project (http://biomonitoring2.org). Sites PAD 03 and 04 are in the Athabasca River Delta and PAD 14 and 33 are in the Peace River Delta. Surficial material in the delta consists of deltaic alluvial deposits and soils, which are mainly silty with some clay, are considered characteristic of prairie wetlands [26]. Field permits were granted by Parks Canada at Wood Buffalo National Park and samplings were conducted by Environment Canada and Parks Canada staff. The field work did not involve endangered or protected species.

### *In silico*–Analysis of Database Sequences

Search strings (S5 Table) were used to query GenBank coverage of vascular plant species for each marker. A taxa list for the local PAD assemblage was compiled from aboveground survey data collected by Parks Canada from 1993–2008 (unpublished monitoring data) and public data from the Alberta Biodiversity Monitoring Institute (accessed October 2013, http://www.abmi.ca/). GenBank coverage of this list was assessed.

Available sequences for the local taxa were downloaded, aligned in MEGA version 6.06 [27] and made into mock sequencing reads by cropping to amplicon regions. Mean interspecific uncorrected pairwise distances were calculated in MEGA [27] using only species with sequences for all four markers. Each species’ minimum interspecific genetic distance (nearest neighbour distance, NND) was extracted from the distance matrix for each locus. Significant differences in NNDs among DNA markers were identified using the Friedman rank sum test and post hoc Wilcoxon signed rank test, treating species as the blocking unit, in R version 3.1.2 [28].

All mock amplicons were searched against the available GenBank sequences for the locus (S5 Table) using megaBLAST version 2.2.25 [29]. A default word size of 28 and minimum cut-offs of 98% identity and 10^−20^ *E*-value were used for *mat*K, *rbc*L, and ITS2 [1,10,14]. Due to the small size of *trn*L sequences, a word size of 12 and minimum cut-offs of 98% identity and 0.1 *E*-value were used to increase number of sequence assignments obtained. Taxonomy was consolidated for all hits tying for top score with conflicts reported as “ambiguous”. Results were compared against the known taxonomy for each sequence to count proportions of correct, incorrect, or ambiguous assignments.

### *In situ*–Analysis of Soil Cores

#### DNA Metabarcoding of Soil Samples

Three soil cores were collected from each of the four sites in August of 2011, 2012, and 2013 except for site PAD 14 in 2012 where only two cores were retrieved. For each of these 12 sampling instances, a 1 m^2^ area was cleared of surface debris and plant material and the soil cores were collected with 10 cm sterile syringes. Soil was subsampled into UltraClean^®^ Soil or PowerSoil^®^ DNA Isolation kit (MO BIO Laboratories; Carlsbad, California, USA) lysis tubes for DNA extraction. Amplicons were prepared using established primer sets for *mat*K, *rbc*L, ITS2, and the *trn*L intron P6 loop (S1 Table) and custom PCR protocols (S2 and S3 Tables). Amplicons were purified with the MinElute^®^ PCR Purification kit (QIAGEN; Toronto, Ontario, Canada) except for *trn*L amplicons due to size limitations. Illumina adaptors were added in a second round of PCR (S3 and S4 Tables) and all amplicons were purified using the MinElute^®^ kit. After indexing, Illumina HTS was performed with either MiSeq Reagent v2 sequencing kits capable of producing 2 x 250 bp sequences (all *trn*L amplicons and PAD 14 and PAD 33 *rbc*L amplicons) or v3 sequencing kits capable of producing 2 x 300 bp sequences (all *mat*K and ITS2 amplicons and PAD 03 and PAD 04 *rbc*L amplicons). Similar sequencing depth was applied to all samples.

Raw sequences for *rbc*L and *mat*K were quality filtered using PRINSEQ version 0.20.2 lite [30] and paired-ends were concatenated. Overlapping paired-end reads for ITS2 and *trn*L sequences were assembled using PANDASEQ version 2.7 [31] and quality filtered with PRINSEQ. For the OTU analysis, sequences were denoised and clustered into OTUs at 98% similarity (95% similarity for ITS2) with USEARCH version 6.0.307 [32]. OTU centroid sequences were searched against available GenBank sequences using megaBLAST with low stringency match parameters (minimum 70% identity and 0.1 *E-*value) to eliminate non-vascular plant OTUs. Alternatively for taxonomic assignments, sequences were denoised with USEARCH and searched against their respective reference databases using megaBLAST with high stringency match cut-offs (described above). A minimum of 10 sequences had to be assigned to any taxonomic group or OTU within a sample to count it as present and OTUs had to a have a minimum of 100 sequences assigned across all samples to be included in analyses.

Molecular protocols, reaction conditions and all parameters used for sequence processing are detailed in S1 Appendix.

#### Recovery—Sequence Output and Filtering

The numbers of sequences per sample were compared at multiple stages of processing. Significant differences in sequence recovery among DNA markers were identified using a randomized block ANOVA test with post hoc Tukey’s test or Friedman rank sum test with post hoc Wilcoxon signed rank test in R [28], treating soil sample as the blocking unit. DNA marker specificity was assessed by comparing median numbers of sequences per sample assigned to groups other than vascular plants (i.e. non-vascular plants, algae, or fungi).

#### Taxonomic Resolution of Recovered Vascular Plant Sequences

Differences in taxonomic resolution were measured based on the proportion of sequences in each sample assigned to vascular plant orders but not assigned at the family, genus, and species levels. Friedman rank sum tests blocked by soil sample were used to test for significant differences in proportions among DNA markers.

#### DNA Marker Complementarity

Differences in overall taxonomic breadth or detection biases were identified by comparing cumulative diversity for the 35 soil cores. DNA marker richness and composition were then compared for pooled sampling replicates (n = 12) at OTU, order, family and genus levels using ANOVA tests blocked by sampling instance and post hoc Tukey’s tests. Compositional agreement among DNA markers at order, family, and genus levels was calculated from Jaccard dissimilarities using “betadisper” in the vegan package (version 2.2–1) in R [33]. This function performed PCoAs on the dissimilarity matrices, identified spatial medians among the four DNA marker points for each sampling instance, and measured the distance of each point to the median. Mean distances were compared among DNA markers using ANOVA tests blocked by sampling instance and post hoc Tukey’s tests to identify if any DNA markers were consistently more dissimilar in their composition estimates from the other markers. All ANOVA tests were performed in R [28].

All raw sequence data is deposited in NCBI’s Sequence Read Archive (SRA Accession SRP073252) under BioProject PRJNA318025.

## Results

### *In silico*–Analysis of Database Sequences

**Fig 1:**
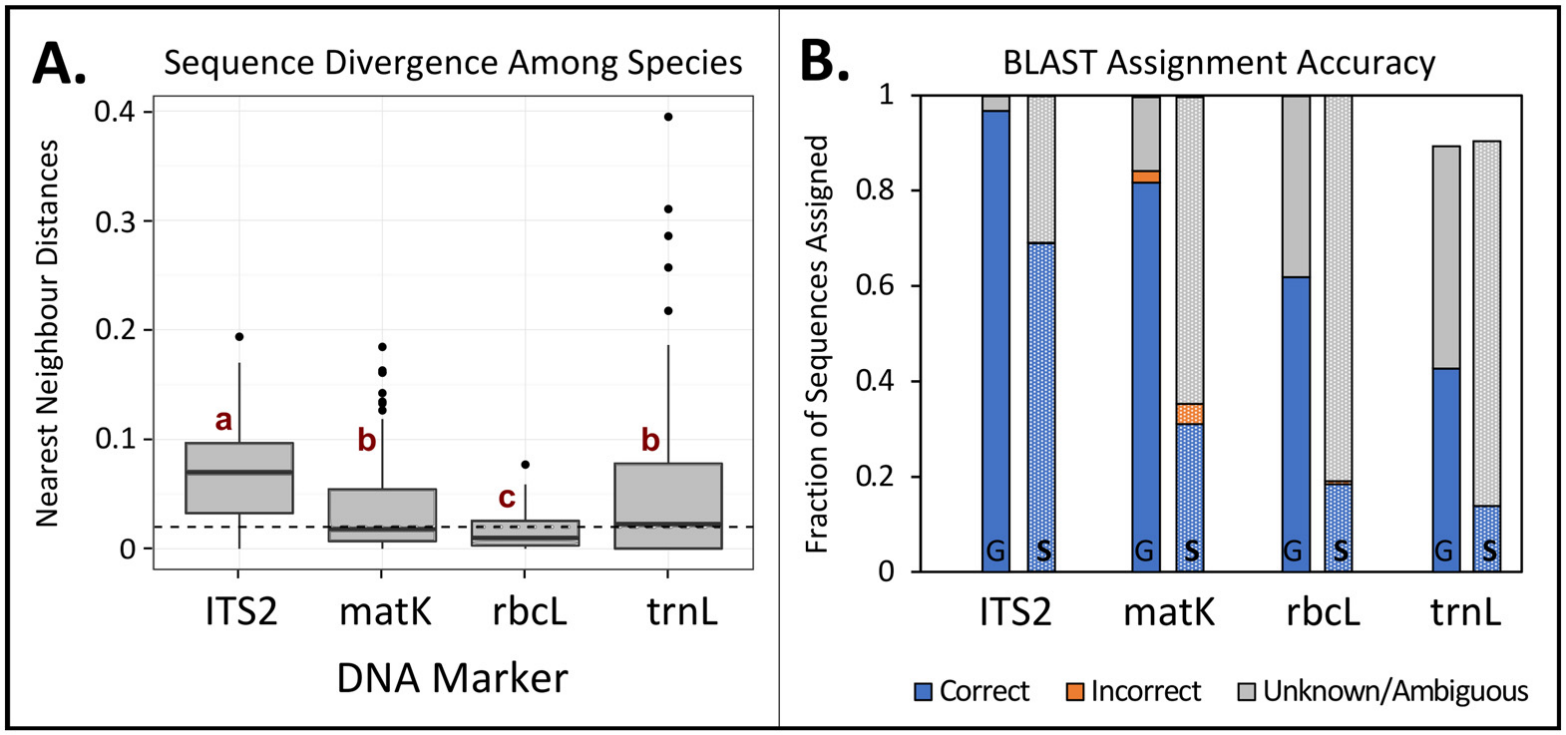
*In silico* comparisons of DNA markers using known database sequences. (A) Nearest neighbour distances provide relative sequence divergence among species (n = 115). Letters denote significant differences (α = 0.05) and the dotted line shows 2% sequence divergence. (B) Associated accuracy of taxonomic assignments of mock sequence reads using BLAST was assessed at the genus (“G”, n = 919, 447, 432, 364) and species levels (“S”, n = 893, 420, 410, 320 for ITS2, *mat*K, *rbc*L, and *trn*L, respectively).

ITS2 had the greatest coverage on GenBank of the four DNA markers in terms of total number of vascular plant species present and ratio of sequences to species. All loci had 94–100% coverage of the 28 orders, 51 families, and 131 genera previously recorded in the study region but the *trn*L intron had only 69% coverage of the 238 known species compared to 81–83% for *rbc*L, *mat*K, or ITS2 (Table 1, but see S2 Appendix for detailed list). Nearest neighbour distances (NNDs) were significantly greater for ITS2 compared with *mat*K and *trn*L while *rbc*L had significantly lower sequence divergence among species (Fig 1A, Table A in S6 Table). Likewise, ITS2 demonstrated the most correct, unambiguous taxonomic assignments of these known sequences followed by *mat*K, *rbc*L, and *trn*L (Fig 1B). The most incorrect assignments occurred with *mat*K while *trn*L showed the most ambiguous or unknown assignments including 10% of sequences with no matches (Fig 1B).

**Table 1:** Sequence database (GenBank) coverage of the four DNA markers summarized for both total entries and the targeted plant list.

	Total database			Targeted PAD vascular plant list			
DNA marker	All species	Vascular plant species	Ratio seq: spp	Order *(n = 28)*	Family *(n = 51*)	Genus *(n = 131)*	Species *(n = 238)*
*mat*K	43,966	43,610	2.03	100%	100%	98%	83%
*rbc*L	44,157	34,331	2.09	100%	100%	98%	82%
ITS2	175,035	75,981	2.34	100%	98%	97%	81%
*trn*L	55,752	51,789	1.83	100%	98%	94%	69%

### *In situ*–Analysis of Soil Cores

#### Recovery—Sequence Output and Filtering

**Fig 2:**
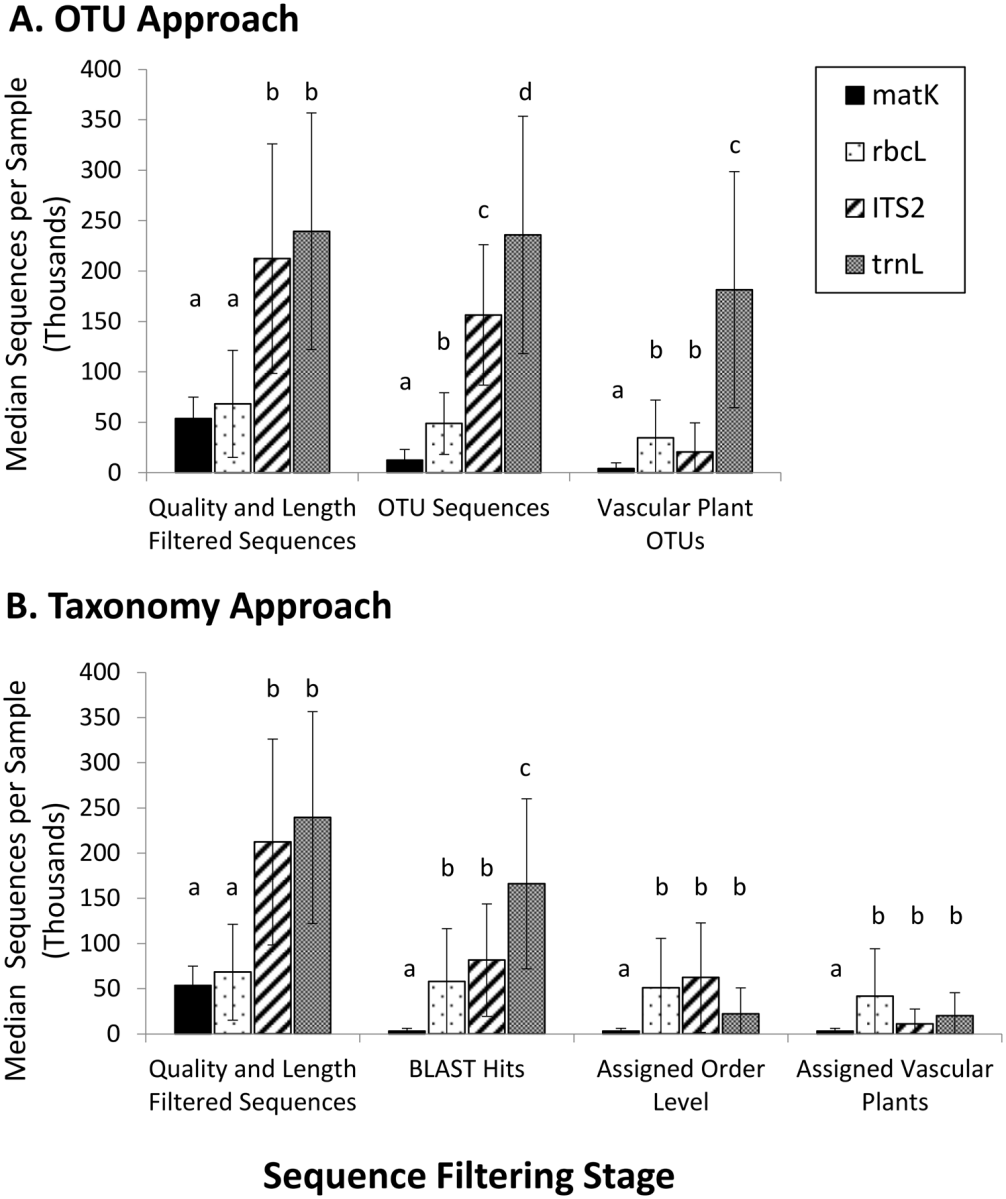
*In situ* sequence recovery by DNA marker. Median number of sequences per soil sample (n = 35) recovered for each DNA marker at sequential stages of filtering in the OTU approach (A) and taxonomy approach (B). Error bars represent median absolute deviations and letters denote significant differences (α = 0.05) at each filtering stage.

Sequences recovered from 35 soil cores collected in the Peace-Athabasca Delta, northern Alberta, Canada were analyzed using operational taxonomic units (OTUs) and taxonomic assignments. There were no significant differences among DNA markers in the number of raw sequence reads per sample but after filtering for quality and length, approximately four times more sequences per sample were retained for ITS2 and *trn*L than *mat*K and *rbc*L (Fig 2). During the OTU analysis, significant DNA marker differences in recovery were identified after all filtering stages (Fig 2A). In total, 1220, 1442, 1781, and 2026 OTUs were identified for *mat*K, *rbc*L, ITS2, and *trn*L, respectively but only 38% of *mat*K OTUs had database matches compared to 77–91% of OTUs for other DNA markers. After filtering to just vascular plant OTUs, *mat*K retained significantly fewer sequences per sample compared to *rbc*L and ITS2 while *trn*L retained significantly more sequences (medians of 4100, 18200, 20700, and 34600 sequences, respectively). These sequences represented totals of 363, 834, 176, and 1071 vascular plant OTUs for *mat*K, *rbc*L, ITS2, and *trn*L, respectively.

DNA marker differences were also found at all filtering stages in the taxonomic assignment approach (Fig 2B). *mat*K had significantly fewest sequences per sample at all stages whereas *trn*L, *rbc*L, and ITS2 did not show significant differences in sequence recovery once assignment results were filtered to order level. After all filtering, medians of 3200, 41700, 11100, and 19900 sequences were assigned to vascular plant orders for *mat*K, *rbc*L, ITS2, and *trn*L, respectively. ITS2 had the lowest specificity of the four markers with a median of 59% of sequences per sample assigned to non-vascular plants (e.g. mosses), fungi and algae. Only the *mat*K sequences were specific to vascular plants while *trn*L and *rbc*L produced medians of 6% and 9% non-vascular plant sequences, respectively. See Table B in S6 Table for statistical test output.

#### Taxonomic Resolution of Recovered Vascular Plant Sequences

**Fig 3:**
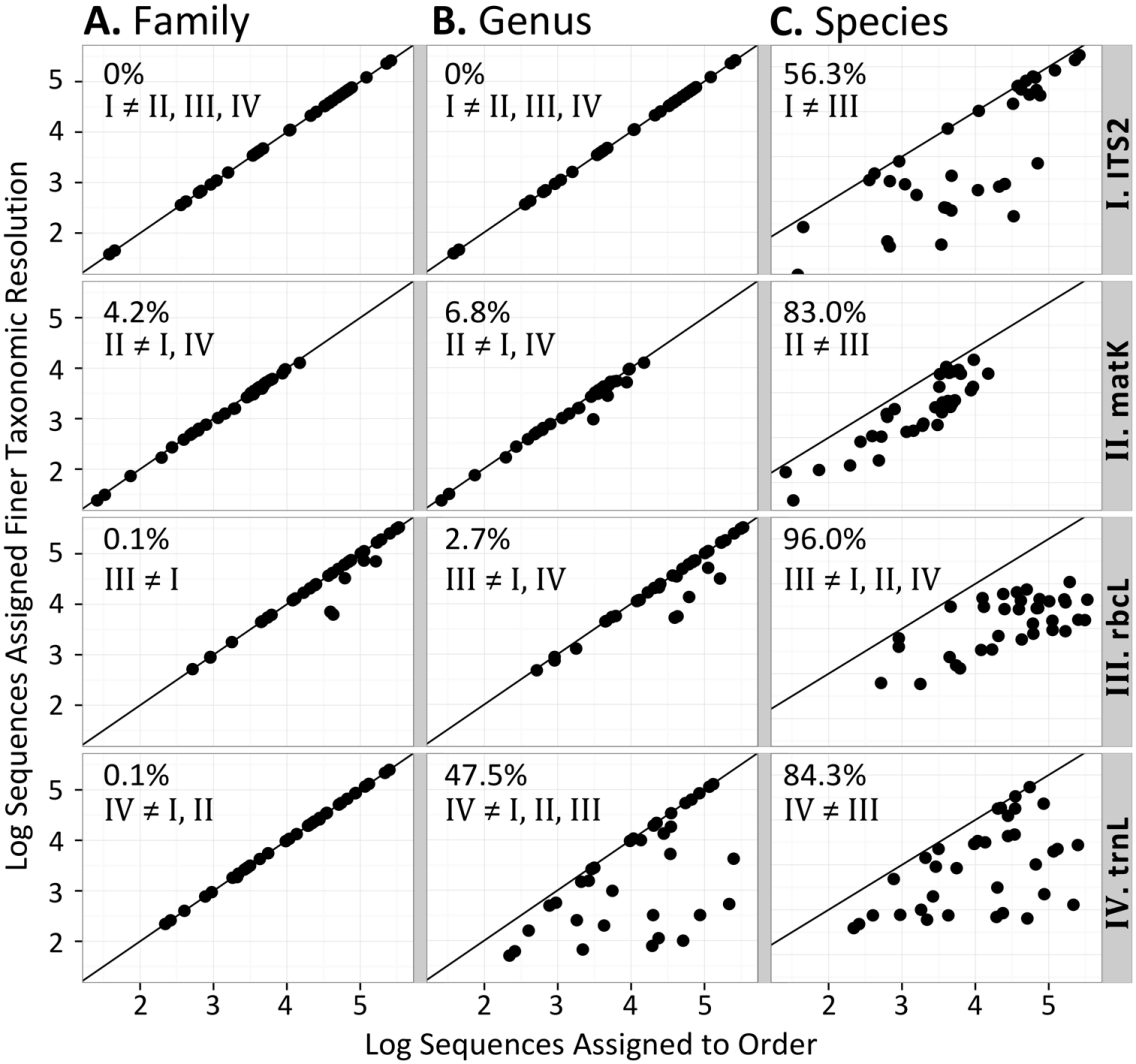
*In situ* taxonomic resolution of sequences. Log number of sequences per sample (n = 35) assigned unambiguously at the family (A), genus (B), or species (C) level versus the log number of sequences assigned at the order level are shown for four DNA markers. Lines indicate the upper limit if all sequences are resolved. Median percent of sequences per sample unassigned at each level are indicated. Roman numerals denote significant marker differences (α = 0.05) in taxonomic resolution.

There were significant differences among DNA markers in the percent of taxonomically unassigned sequences below order level (Fig 3, Table C in S6 Table). All ITS2 sequences were unambiguously assigned family and genus identities. At the genus level, a significantly greater proportion of *trn*L sequences were unassigned compared to the other DNA markers (median of 47.5% versus 0–6.8% unassigned). At the species level, all markers showed noticeably low sequence assignment but *rbc*L was the most affected and significantly different from other DNA markers (median of 96.0% versus 56.3–84.3% unassigned). Due to such low proportions of sequences assigned unambiguously to species, only results for order, family, and genus levels are discussed further.

#### DNA Marker Complementarity

**Fig 4:**
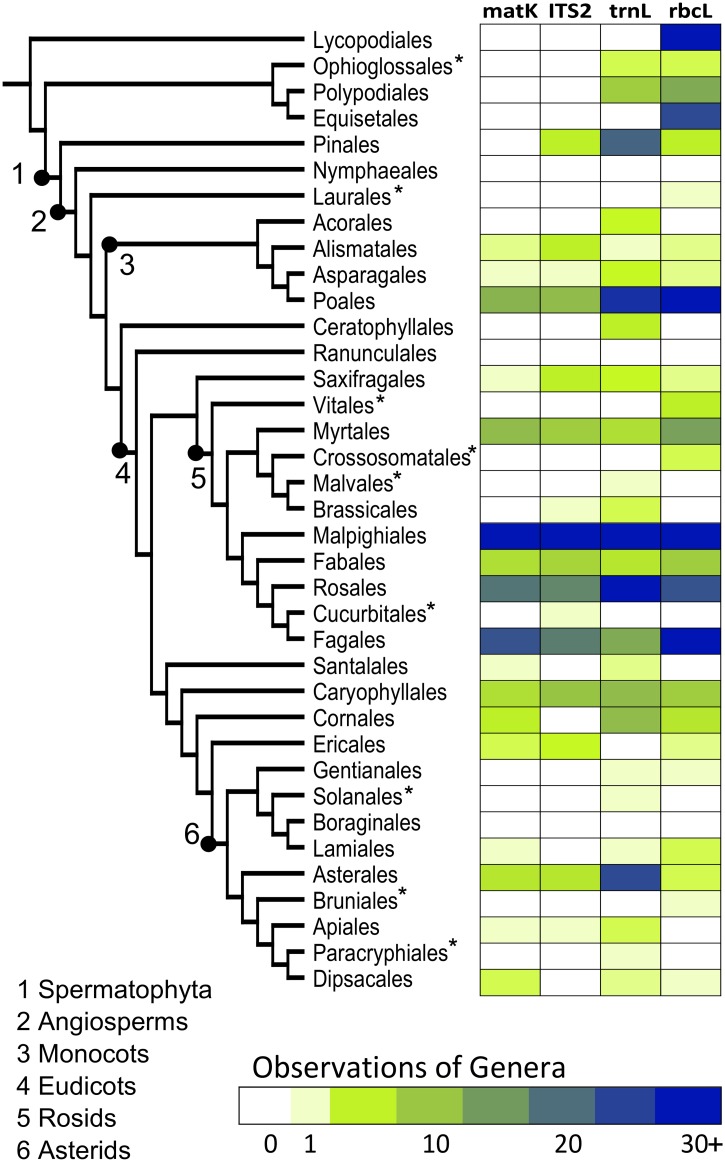
Complementarity of plant diversity reported *in situ* by the four DNA markers. Number of observations of vascular plant genera from 35 soil cores are grouped by order and arranged by established phylogenetic relationships [34,35]. Larger plant clades are labeled and orders that were not previously recorded in surveys are indicated with asterisks.

Following the taxonomic assignment analysis, a total of 36 orders, 63 families, and 142 genera were detected in the 35 soil samples across all four DNA markers. Taxa lists for ITS2 and *mat*K were highly overlapping with lists from past vegetation surveys while *rbc*L and *trn*L had greater numbers of taxa not observed in previous surveys (Table 2). The total compositional overlap, taxonomic breadth, and any major taxonomic biases of the four DNA markers can be seen in Fig 4. All orders observed using *mat*K were also observed with at least one other DNA marker and *mat*K only detected angiosperm groups. ITS2 was also highly overlapping with the other DNA markers because all orders were also observed with other DNA markers except for one order (Cucurbitales), represented by a single observation of a single genus, *Cucumus*, which includes primarily cultivated species. Only seed bearing vascular plants (Spermatophyta) were detected with ITS2. The other two DNA markers, *rbc*L and *trn*L, both had observations of genera from multiple unique orders and included both seed bearing and seedless vascular plant orders. In particular, only *rbc*L reported observations of horsetails (Equisetales) and club mosses (Lycopodiales). Rosids showed similar numbers of observations across all four DNA markers whereas *Poales* genera were more frequently observed with *rbc*L and *trn*L. As well, *trn*L showed increased observations of Asterids and gymnosperms while *rbc*L had the most observations of seedless vascular plant genera.

**Table 2:** Total numbers of vascular plant taxa that were observed across 35 soil cores with eDNA and overlap with the list of previously recorded taxa, given the database coverage.

		# Plant taxa		
Level	Locus	eDNA	Veg^1^	DB^2^
**Orders**	**Total**	**36**	**27**	**27**
	*rbc*L	27	21	27
	*mat*K	17	17	27
	ITS2	16	15	27
	*trn*L	28	24	27
**Families**	**Total**	**63**	**36**	**36**
	*rbc*L	42	23	36
	*mat*K	22	21	36
	ITS2	20	19	35
	*trn*L	43	32	36
**Genera**	**Total**	**142**	**56**	**56**
	*rbc*L	79	32	56
	*mat*K	37	33	56
	ITS2	34	28	53
	*trn*L	69	32	54

^1^ Number of taxa detected with eDNA known from prior aboveground vegetation surveys in the delta;

^2^ Database coverage of these previously recorded taxa for each respective marker.

To assess marker agreement in site-level vascular plant diversity, we pooled soil core replicates for the 12 sampling instances. Average site-level *mat*K and ITS2 OTU richness was significantly less than *rbc*L and *trn*L OTU richness (means of 39, 37, 133, and 217 OTUs, respectively). Similarly, in the taxonomic assignment approach, mean site-level *mat*K or ITS2 richness was significantly less than *rbc*L or *trn*L richness at order, family, and genus levels (means of 4.8, 5.7, 8.7, or 10 orders, 5.2, 5.8, 10.1, or 11.7 families, and 6.7, 6.4, 15.3, or 13.3 genera, respectively) (Table D in S6 Table). Looking at site-level vascular plant composition, there were no significant differences among DNA markers in mean distance to sampling instance spatial median in the Principal Coordinates Analyses (PCoAs) based on Jaccard dissimilarities at order or family level. At the genus level, however, mean *trn*L PCoA distance was significantly greater than mean ITS2 and *mat*K distances and mean *rbc*L PCoA distance was significantly greater than mean *mat*K distance but intermediate to ITS2 or *trn*L mean distances (Table E in S6 Table). Greater distances suggest greater dissimilarity in site-level vascular plant composition reported by these DNA markers.

## Discussion

### *In silico*–Analysis of Database Sequences

DNA barcoding relies on database completeness and whether entries are both correct and informative [36]. For example, although *Sagittaria cuneata* is a common species in the study region, there was no reference sequence available for this species for any of the four DNA markers (S2 Appendix) rendering metabarcoding identification impossible. Nine of the 238 taxa previously recorded in the PAD region lacked reference sequences for all four DNA markers and thus could not have been identified in the soil samples. An additional 13 species were only represented in the database by one of the four loci which means that those species could have only been correctly identified if recovered and resolved by that particular DNA marker. Even though OTU approaches can be used to measure the diversity represented by a single DNA marker and avoid the limitations of annotation [37], taxonomic assignment is necessary to link data to established monitoring indices such as the florist quality index (e.g. [38]) and other current standard practices.

In our study, *trn*L had distinctly more total database gaps than the other three loci. Database coverage, however, was essentially complete across the four loci for the previously recorded taxa that were subsequently observed *in situ* by at least one of the DNA markers (Table 2) suggesting that database gaps were not the main limitation for any particular DNA marker for the *in situ* analysis of soil eDNA. Instead, this indicates that DNA marker differences observed in the analysis of soil samples were likely due to differences in overall database quality, sequence recovery, or sequence resolution.

Trends in NNDs were consistent with previous reports of sequence resolution among the four markers [4,16–18,39] with nuclear ITS2 showing the highest level of sequence divergence, hence, providing least amount of assignment ambiguity. Differences in plastid versus nuclear evolutionary dynamics may underlie differences in species discrimination of the four loci [39] and confirm that a nuclear locus is necessary to increase species-level resolution for plant biodiversity assessments [18].

### *In situ*–Analysis of Soil Cores

#### Recovery—Sequence Output and Filtering

While number of raw sequences recovered were not statistically different across loci, non-overlapping paired-end reads (i.e. *mat*K and *rbc*L) showed lower sequence retention following quality and length filtering compared to overlapping paired end reads (i.e. *trn*L and ITS2). Sequence quality declines towards the 3’ end of reads and the longer amplicons do not have added support from overlapping regions [31]. Since *mat*K subsequently had the fewest sequences returned with database matches, it is likely that 90% of high quality *mat*K sequences represented sequencing or PCR artifacts. Poor PCR success has been previously noted for *mat*K [16] and continues to be an important concern for DNA metabarcoding. Contrary to *mat*K, the majority of *rbc*L sequences passing quality filters also returned database hits. These *rbc*L sequences were dominated by the targeted vascular plant sequences even though non-vascular plant and algal sequences were also present. These additional sequences could potentially be used for surveys of lower plants and algal taxa from soil eDNA.

Less than half of good quality ITS2 sequences returned database hits with the high stringency search parameters (Fig 2B) and this may reflect the increased intragenomic and intraspecific variability of the region despite the relatively high database coverage [17,37]. Predictably, a much larger proportion of sequences were retained for ITS2 in the low stringency search for OTU analysis. ITS2, however, had the lowest specificity because the majority of sequences belonged to non-target groups. The primers used here showed a propensity to amplify fungal sequences. This is likely due to relatively few nucleotide differences among fungal and plant lineages in the conserved regions used for the primer binding sites [40]. Also, algal ITS sequences in the database are sometimes misidentified as fungi and vice-versa [40] so it is possible that some of the ITS sequences identified as fungi here constituted mislabelled plant sequences.

Although *trn*L had the most sequences returned with database matches, the majority of those were not assigned taxonomy at the minimum order level (Fig 2B). On further investigation, this was partly attributed to a few common sequences having “Uncultured Streptophyta clone” among their equally scoring top database hits obscuring what would have been a family level identification to Salicaceae. Other *trn*L sequences were assigned to this family in each sample so this was not expected to affect overall diversity reported, however, improved curation of the reference database could aid recovery. Since *trn*L had good specificity with the majority of sequences belonging to vascular plants but poor annotation and taxonomic resolution, it had greater recovery following the OTU approach. In summary, ITS2, *rbc*L and *trn*L showed similar magnitudes of overall sequence recovery while *mat*K had significantly lower sequence recovery.

#### Taxonomic Resolution of Recovered Vascular Plant Sequences

Taxonomic resolution is critical for biomonitoring [2,10]. ITS2 had the best taxonomic resolution of all loci with all sequences assigned to an order also unambiguously assigned to a family and genus as well as the most species level identifications. This is in line with previous observations [18]. *mat*K and *rbc*L also showed relatively high taxonomic resolution through to genus level but lacked optimal species-level resolution as previously noted [16]. In contrast, large proportions of *trn*L sequences were only resolved to family level in agreement with findings from the original study [21]. Since *trn*L was shown to have somewhat greater sequence divergence within our local taxa, this relatively lower taxonomic resolution was due to either annotation difficulties (e.g. database entries missing full taxonomic identifications), a lack of sequence divergence outside of taxa included in the NND test (overestimated divergence), or biased sample composition towards taxa that are less resolved with this DNA marker.

#### DNA Marker Complementarity

In our analyses *rbc*L and *trn*L consistently reported greater overall richness values compared to *mat*K and ITS2. This is in contrast to the study by Yoccoz, *et al*. [12] that found significantly greater sequence recovery and OTU diversity for *trn*L than *rbc*L. These loci both showed greater taxonomic breadth within vascular plants suggesting that more unique taxa were detected as compared to *mat*K and ITS2. For example, *rbc*L detected common lower plants such as club mosses and horsetails that the other loci missed which may account for some of the increased richness observed. Additionally, lower *matK* and ITS2 richness might be due to lower recovery of target taxa for these markers. Suboptimal *mat*K primer binding may have impeded maximal recovery of vascular plants whereas lack of specificity of ITS2 primers resulted in sequencing throughput shared with fungal and algal species.

Dissimilarity in taxa reported by different loci increased at finer scales such that genus level plant diversity showed less marker agreement than at order level. In our study, *trn*L, and to some degree *rbc*L, showed significantly greater PCoA distances compared to *mat*K and ITS2 only at the genus level indicating less congruence in the reported plant composition. Two DNA markers may be seen as more dissimilar if they detect largely different numbers of taxa or if they detect distinct groups of taxa. Since *rbc*L and *trn*L showed significantly greater richness than *mat*K and ITS2, the decreased congruence in the reported plant diversity with these two DNA markers could be due to the added information from their increased taxonomic breadth rather than just a lack of overlap with the other DNA markers.

It is important to consider the overall quality and accuracy of community profiles reported through DNA metabarcoding and address potential sources of error outside of recovery, resolution, and annotation. Nine vascular plant orders previously not recorded in the region were observed with at least one DNA marker (Fig 4), many of which are unlikely to be native to a boreal wetland. Most of these groups, however, include economically important and commercially traded species (e.g. crops, ornamentals, timber, etc.) and are represented by a single observation with just enough sequences to pass our filters. Due to the sensitivity of HTS, there are many ways trace DNA from species in these groups could enter the samples in the field or during handling in lab. For example, it is known that extraction kits and other reagents used in the lab are not always DNA-free [41,42]. Furthermore, not all false positives are caught during data filtering which may have inflated the eDNA values in Table 2. Interpretation of metabarcoding output continues to advance and new research suggests that occupancy models will improve detection of false positives resulting from sequencing artefacts or sample contamination compared with rule-of-thumb filtering (i.e. static thresholds for number of sequences needed to make an identification) applied here [43].

Another option to limit false positives is to search against a geographically constrained database with only the known local flora [11,12,44] but this prevents the observation of novel or unexpected taxa (e.g. invasive species) that are present. In this study we wished to test how the four DNA markers would perform with no assumptions about what taxa would be found and no manual filtering of select taxa. For example, Ophioglossales was not on the regional vegetation lists obtained for the delta but detected by both *rbc*L and *trn*L at the same site. This group of small seedless vascular plants was likely present but missed by aboveground surveys and would have been excluded from the eDNA survey if a database of only previously recorded taxa had been used. In practice, further refinement of data filtering approaches will help reduce eDNA identification error rates.

### Conclusions

Given the criteria of recovery, annotation, and resolution as well as complementarity of the vascular plant composition identified with different DNA markers, ITS2 and *rbc*L are better choices for performing biodiversity assessments of plants from soil eDNA. The DNA marker *mat*K had the lowest recovery, did not detect unique taxa, and had the lowest taxonomic breadth. The *trn*L P6 loop offered the least taxonomic resolution of recovered vascular plant sequences, either due to low sequence divergence or poor annotation, and it showed the least similarity among the four markers in vascular plant composition within sites at the genus level. Consequently, the *trn*L P6 loop may be more suitable for studies where analysis of only OTUs with limited taxonomic information is sufficient. It also may be more suitable for biodiversity assessment from eDNA when curated databases for local assemblages are already established because this would reduce ambiguities in taxonomic assignments [12,21]. However, these localized reference databases would likely improve taxonomic annotation for any DNA marker.

ITS2 offered superior taxonomic performance despite lower specificity towards vascular plants and improved primer design and optimization of PCR conditions could help address ITS2 specificity issues for future eDNA surveys from soil samples where both plant and fungal DNA is abundant [40]. While *rbc*L had the greatest taxonomic breadth across vascular plants owing to good recovery and annotation, ITS2 complements this with its greater taxonomic depth (resolution) within the seed bearing vascular plants. By using multiple markers, overlap in the observed plant diversity can provide increased support for findings. A multiple marker approach will also increase probability of recovering, resolving, and annotating all taxa in a sample because even if multiple primer sets or degenerate primers are used for a single locus to improve recovery, some taxa may not resolve or lack database coverage with the chosen marker. ITS2 and *rbc*L belong to different linkage groups which can aid in resolution, and both are supported by ongoing reference database development through global Barcode of Life initiatives.

The introduction of HTS-based DNA metabarcoding has been accompanied by promotion of new, non-standard markers or design of new primers for established DNA markers to suit specific taxonomic groups or geographically defined communities of interest [11,20,22,23]. However, the process of *in silico* marker selection and *in vitro* optimization and validation on a case-by-case basis adds time consuming extra steps and detracts from the prospective increase in efficiency of metabarcoding for large-scale biomonitoring. If non-DNA barcode loci are chosen, reference database coverage is much more likely to be a limiting factor in an assessment and introduces the added time and cost of building the required database for each new marker and set of taxa. Furthermore, comprehensive prior knowledge of all local taxa is needed in order to build an effective reference database.

It has been argued that new markers are needed for metabarcoding because established DNA markers like plant DNA barcodes are too long and cannot be recovered from eDNA due to degradation [12,20,22]. We were able to generate full length amplicons for *mat*K, *rbc*L and ITS2 (ranging from 400 to 900 bp) directly from the soil samples using the standard primer sets. The second longest marker (*rbc*L) reported site richness on par with the shortest marker (*trn*L) indicating that marker length within this size range is not a major restriction for soil eDNA. Recent DNA metabarcoding diet analysis of grasshoppers using *rbc*L further reinforces this point [14]. While shorter DNA markers are needed for ancient DNA, biomonitoring or other questions of contemporary biodiversity will benefit from the improved taxonomic resolution offered by the full length DNA barcodes which can be recovered with similar efficiency from samples.

Overall, this study’s findings suggest that plant DNA barcode regions *rbc*L and ITS2 are most suitable for biodiversity assessment of vascular plants from soil eDNA. Our work supports the collaborative development and application of DNA barcoding and metabarcoding rather than treating them as two distinct methodologies to develop independently.

## Supporting Information

S1 Appendix Metabarcoding methodology. (DOCX)

S2 Appendix Previously recorded taxa and associated database coverage. (XLSX)

S1 Table Primer sequences and expected amplicon sizes for each locus. (DOCX)

S2 Table Optimized PCR conditions used for first round amplification of each locus. (DOCX)

S3 Table Thermocycler programs used with each locus for first and second rounds of amplification. (DOCX)

S4 Table Optimized PCR conditions for amplification of each locus with Illumina tailed primers. (DOCX)

S5 Table Search criteria used to build reference databases for each locus from NCBI's GenBank. (DOCX)

S6 Table Statistical test output for all analyses. (DOCX)
