# Supplementary material for: Large-Scale Monitoring of Plants through Environmental DNA Metabarcoding of Soil: Recovery, Resolution, and Annotation of Four DNA Markers
Source: PLoS One. 2016 Jun 16;11(6):e0157505. doi: 10.1371/journal.pone.0157505 (PMC4911152; doi:10.1371/journal.pone.0157505)
Supplement: S1 Appendix — (DOCX) [file pone.0157505.s001.docx]

# Supporting Information – Appendix 1

# Metabarcoding Methodology

Sample Collection

As part of the Biomonitoring 2.0 project, three soil samples were collected for DNA analysis from each of the four sites (PAD 03, 04, 14, and 33) by a sampling team from Environment Canada in August of 2011, 2012, and 2013 except for site 14 in which only two samples were collected for August 2012. Surface debris and plant materials were cleared inside a square meter and then sterile syringes were used to collect three 10 cm soil cores evenly spaced in an equilateral triangle within this square (subsamples denoted as X, Y, and Z). This area was marked with a pin so that the location within the site could be re-sampled in subsequent years. Each core was transferred to a labelled 50 mL tube and frozen for transportation. Once received, the 35 samples were stored at -80°C until ready for processing.

Subsampling

Samples were thawed at 4°C prior to sub-sampling. Workbenches were wiped down with 70% ethanol, ELIMINase® (Decon Laboratories; King of Prussia, Pennsylvania, USA) and then deionized water before and after all lab procedures. The outside of each 50 mL tube containing a soil core was wiped down with ELIMINase® and deionized water to remove any potential contaminants. A scoopula, also cleaned with ELIMINase® and deionized water, was dipped in ethanol, flamed and let cool briefly before being used to break apart the soil core within the tube. From a single core, soil was added to three lysis bead tubes from the DNA extraction kit until they were approximately two thirds full. This was repeated for all soil cores, flaming the scoopula in between each core. The bench was wiped down and gloves as well as any labware were changed in between samples from different sites and years.

DNA Extraction

DNA was extracted from the 2011 and 2012 soil samples using UltraClean® Soil DNA Isolation kits (MO BIO Laboratories; Carlsbad, California, USA) while the 2013 soil samples were extracted using PowerSoil® DNA Isolation kits (MO BIO Laboratories; Carlsbad, California, USA) which replaced the discontinued UltraClean® kit. Extractions followed kit specifications except, when using the UltraClean® kit, twice the specified volume of the first two reagents were added to the lysis bead tubes. The supernatent from each bead tube was divided over two 1.5 mL tubes for the next steps, resulting in six extractions per sample. The DNA eluted from the six UltraClean® reactions (50 μL each) or three PowerSoil® reactions (100 μL each) was pooled for each sample for a total volume of approximately 300 μL of DNA. Only samples from the same site and year were extracted at the same time to reduce risk of cross-contamination and extraction negatives were made for each round of extraction with the 2013 samples. Samples were labelled and stored at -20°C.

PCR Amplification

During amplicon preparation, samples went through two rounds of PCR amplification for each of the four DNA marker regions. First with unmodified primers to target and amplify the desired locus, then with Illumina-tailed primers that added the adapter needed for sequencing. Established universal plant primers from the Canadian Centre for DNA Barcoding (CCDB) protocols (<http://www.ccdb.ca/resources.php>) were chosen for *mat*K, *rbc*L, and ITS2 amplification and the Taberlet *et al*., 2007 [1] primers were chosen for amplification of the P6 loop of the *trn*L intron. Primer molecules were made by Integrated DNA Technologies (Coralville, Iowa, USA) and their details are summarized in SI Table 1. All amplification reactions were performed in 25 μL total volumes. To minimize risk of cross-contamination, all reactions were prepared in AirClean® 600 PCR Workstations (AirClean Systems; Oakville, Ontario, Canada) using strip tubes with individually attached caps (Eppendorf; Mississauga, Ontario, Canada) and two negative controls were made for every PCR master mix. Test reactions with samples were performed for each DNA marker to optimize annealing temperatures and reaction conditions for mixed template reactions.

First round amplifications were performed in triplicate for each sample to help reduce effects of PCR bias and stock DNA was diluted to 1/10 with HyClone® HyPure^TM^ Molecular Biology Grade Water (GE Healthcare; Logan, Utah, USA) prior to amplification to counteract effects of PCR inhibitors common in soil DNA extracts. The master mixes used for each locus are outlined in SI Table 2. Samples were run for 30 cycles using Mastercycler® pro S thermocyclers (Eppendorf; Mississauga, Ontario, Canada) and the programs for each DNA marker are outlined in SI Table 3.

Gel electrophoresis was used to check the PCR products and negatives. A 1.5% agarose gel was made with 1.5 g of UltraPure^TM^ agarose (Life Technologies; Burlington, Ontario, Canada), 100 mL of 1x Tris-Borate-EDTA (TBE) buffer (Fisher Scientific; Ottawa, Ontario, Canada) and 3 μL of 1% ethidium bromide solution (Fisher Scientific; Ottawa, Ontario, Canada). Wells were loaded with 5 μL of sample and 3 μL of loading dye and 3 μL of 100 bp DNA Ladder (New England BioLabs; Whitby, Ontario, Canada) was used as the size standard. The gel was then run at 150 V for 30 min in TBE buffer and imaged using a transilluminator and DSLR camera. If samples did not display a band of expected size on the gel under these amplification conditions, they were re-run with increased template DNA, primer, magnesium chloride, or some combination of these for up to 40 cycles.

Following successful first round amplification, replicates of *mat*K, *rbc*L, and ITS2 were pooled and purified using the MinElute® PCR Purification kit (QIAGEN; Toronto, Ontario, Canada). Purified DNA was eluted with 30 μL of molecular biology grade water when done by hand or with 15 μL of water when using the QIAcube automated system (QIAGEN; Toronto, Ontario, Canada). Since the *trn*L P6 loop amplicons were expected to be under the minimum size required for purification, these replicate reactions were pooled but not purified.

The Illumina adapters were added next in a second PCR and for this round only one reaction was done per sample for each DNA marker. Master mixes for these reactions are summarized in SI Table 4. For *mat*K, two different mixes were used because half of the samples successfully amplified using the same reaction conditions from the first PCR while others showed better amplification using the same master mix as *rbc*L and ITS2 but with 3 mM MgCl_2_. Samples were run for 15 cycles on the thermocyclers using the same programs as before, however, the annealing temperature for *mat*K was raised to 50°C for any samples run using the second master mix. The samples as well as reaction blanks and negative controls carried from previous steps were checked using gel electrophoresis. Any samples that failed to produce clear bands of expected size were re-run with increased template, primer or both for up to 20 cycles. All samples were then purified with the MinElute® PCR Purification kit and eluted with either 15 or 30 μL of molecular biology grade water.

Library Preparation and Sequencing

Samples to be pooled in the same sequencing run underwent dual indexing. The indices were added through an additional 12 cycle PCR with index primers that bind to the Illumina adapter sequence in each sample. Indexing was completed according to Illumina kit specifications and each reaction contained 1x PCR buffer (Life Technologies; Burlington, Ontario, Canada), 2 mM MgCl_2_ (Life Technologies; Burlington, Ontario, Canada), 0.2 mM dNTPs (Kapa Biosystems; Wilmington, Massachusetts, USA), 0.02 μM of forward and reverse index primer (Illumina; San Diego, California, USA), and 0.1 U/μL Platinum® Taq DNA polymerase (Life Technologies; Burlington, Ontario, Canada), with 2 μL of purified, adapter ligated amplicon.

After indexing, samples were quantified using the Quant-iT^TM^ PicoGreen® dsDNA Assay kit (Life Technologies; Burlington, Ontario, Canada) with a BS-380 Mini-Fluorometer (Turner BioSystems; Sunnyvale, California, USA) and then the individual libraries for a single sequencing run were pooled in equimolar amounts. The Agencourt AMPure^TM^ XP system (Beckman Coulter; Mississauga, Ontario, Canada) was used to purify this combined library and remove short DNA fragments or dimers. The pooled and purified library was quantified again with the Quant-iT^TM^ PicoGreen® dsDNA Assay kit and the length distribution of DNA fragments was obtained using the DNA 7500 kit for the 2100 Bioanalyzer (Agilent Technologies; Santa Clara, California, USA) in order to estimate the number of DNA molecules in solution. The library was diluted to 2 nM if being prepared for a MiSeq Reagent v2 sequencing kit or to 4 nM if being prepared for a MiSeq Reagent v3 kit (Illumina; San Diego, California, USA) and then 5% PhiX, the internal standard, was added.

Sequencing was spread across a total of four MiSeq runs but always done in combination with samples from other projects such that a total of approxi*mat*ely 65 samples were sequenced each run. The *trn*L amplicons were all sequenced using MiSeq Reagent v2 kits and the ITS2 and *mat*K amplicons were all sequenced with MiSeq Reagent v3 kits. The *rbc*L amplicons for PAD 14 and 33 were sequenced with a MiSeq Reagent v2 kit while the *rbc*L amplicons for PAD 03 and 04 were sequenced with MiSeq Reagent v3 kits. Sequencing was completed according to the manufacturer’s protocol for the respective reagent kit.

Sequence Processing

The general bioinformatics pipeline for processing raw sequences was to pair sequences, filter for quality and length thresholds, and then dereplicate files to cluster identical sequences. After some additional denoising, sequences were either clustered into operational taxonomic units (OTUs) or searched against a reference database to obtain taxonomic information. Initial base calling and separation of sequences by index was completed with the built-in MiSeq Control Software, version 2.3.0.3 (Illumina; San Diego, California, USA). For pairing and quality filtering, different protocols were established depending on the degree of sequence overlap expected for each locus: no overlap (*mat*K and *rbc*L), partial overlap (ITS2), or complete overlap including primers (*trn*L intron P6 loop).

When no overlap was expected, sequences were quality and length filtered using PRINSEQ version 0.20.2 lite [2] before pairing. In this case, primers were trimmed from 5’ ends of *mat*K and *rbc*L sequences, the 3’ ends were trimmed using a sliding window of 10 bp with steps of 5 bp to remove sequence where bases have Phred scores less than 20, and any sequences shorter than 150 bp after trimming were removed. Due to the possibility of a few bases of overlap in *rbc*L sequences obtained with the MiSeq Reagent v3 kit, these sequences were also trimmed to a maximum length of 270 bp. The reverse sequences were then reverse complemented using the seqtk package (https://github.com/lh3/seqtk) and then concatenated to the end of the corresponding forward sequence for a minimum length of 300 bp. Due to sequence quality score deterioration at 3’ ends stemming from an overabundance of short non-target fragments present in the library containing all *mat*K amplicons, the 2013 *rbc*L amplicons for PAD 3 and 4, and the 2011 *rbc*L amplicons for PAD 3 X and Z, these files were quality filtered with a less stringent Phred score of 10.

For the partially overlapping ITS2 sequences, PANDASEQ version 2.7 [3] was used to pair forward and reverse reads using the PEAR algorithm [4] with a minimum overlap of 50 bp. Primers were also trimmed and any sequences with N’s were removed. The paired sequences were then quality trimmed using PRINSEQ with the same sliding window as before and removing any sequences less than 200 bp and any over 500 bp.

The *trn*L P6 loop amplicons were smaller than a single read length and each direction was expected to contain both forward and reverse primers. PANDASEQ was again used to pair forward and reverse reads. The default algorithm was used with a minimum overlap of 10 bp, primers were trimmed after pairing, and any sequences with N’s were removed. Paired sequences were also quality trimmed with PRINSEQ using the sliding window and then any sequences shorter than 10 bp or longer than 150 bp were discarded.

All paired and quality filtered sequences were then converted to FASTA format and remaining uncalled bases were removed if necessary.

For the reference database searches, *rbc*L, *mat*K, and ITS2 sequences were sorted by decreasing length, clustered in USEARCH version 6.0.307 [5] at 99% identity for denoising, and then chimeras were removed with the de novo UCHIME algorithm [6]. Due to their small size, the *trn*L intron sequences only underwent full length dereplication to remove exact duplicates with no further denoising required. Cluster centroid sequences were queried against their respective databases (SI Table 5) using megaBLAST version 2.2.25 [7]. The megaBLAST search for *mat*K, *rbc*L, and ITS2 was run using the default word size of 28 and reported hits with a minimum 98 percent identity and E-value threshold of 10^-20^. Due to the small size of the *trn*L P6 loop sequences, megaBLAST was run using a word size of 12 and E-value threshold of 0.1 with the minimum of 98 percent identity. Taxonomy was retrieved and reported for the hits tying for top score with any conflicts reported as “ambiguous”. The taxonomy output was filtered to only retain sequences assigned to vascular plant orders based on a match covering either 90% of query length for variable length ITS2 and *trn*L or a minimum 150bp for *mat*K or *rbc*L which have a central gap and only report the length of a contiguous match. A minimum of 10 sequences had to be assigned to any taxonomic group within a sample to count it as present.

For the molecular diversity or OTU approach, FASTA files for each sample were dereplicated, sorted by decreasing length, and then clustered at 98.5% identity using USEARCH version 7.0.1090 [5]. Since *mat*K and *rbc*L were expected to have internal gaps as artifacts of the sequencing process, parameters were set to not count internal gaps against the identity score for these loci. Chimeric sequences were removed with the de novo UCHIME algorithm and any remaining singletons were also removed. Cluster centroid sequences from all soil samples were pooled and then clustered again at 98% identity to create the OTUs for each DNA marker. The ITS2 sequences were clustered at 95% due to higher levels of expected within species sequence variation [8] and significantly greater interspecific distances. A summary matrix was generated showing OTU membership across samples with a minimum total cluster size of 100 and a minimum of 10 sequences required within a given sample to count the OTU as present. The centroid sequences for these OTUs were then searched against their respective GenBank databases using low stringency match criteria of 70% identity and an *E*-value of 0.1 in order to exclude non-target sequences.

References

1. Taberlet P, Coissac E, Pompanon F, Gielly L, Miquel C, Valentini A, et al. Power and limitations of the chloroplast trnL (UAA) intron for plant DNA barcoding. Nucleic Acids Res. 2007 Feb;35(3).
2. Schmieder R, Edwards R. Quality control and preprocessing of metagenomic datasets. Bioinformatics. 2011 Mar 15;27(6):863-4.
3. Masella AP, Bartram AK, Truszkowski JM, Brown DG, Neufeld JD. PANDAseq: Paired-end assembler for illumina sequences. BMC Bioinformatics. 2012;13:31.
4. Zhang J, Kobert K, Flouri T, Stamatakis A. PEAR: a fast and accurate Illumina Paired-End reAd mergeR. Bioinformatics. 2014 Mar 1;30(5):614-20.
5. Edgar RC. Search and clustering orders of magnitude faster than BLAST. Bioinformatics. 2010 October 1, 2010;26(19):2460-1.
6. Edgar RC, Haas BJ, Clemente JC, Quince C, Knight R. UCHIME improves sensitivity and speed of chimera detection. Bioinformatics. 2011 Aug 15;27(16):2194-200.
7. Zhang Z, Schwartz S, Wagner L, Miller W. A greedy algorithm for aligning DNA sequences. J Comput Biol. 2000 Feb-Apr;7(1-2):203-14.
8. Song JY, Shi LC, Li DZ, Sun YZ, Niu YY, Chen ZD, et al. Extensive pyrosequencing reveals frequent intra-genomic variations of internal transcribed spacer regions of nuclear ribosomal DNA. PloS one. 2012 Aug;7(8).
