# Supplementary material for: Large-Scale Monitoring of Plants through Environmental DNA Metabarcoding of Soil: Recovery, Resolution, and Annotation of Four DNA Markers
Source: PLoS One. 2016 Jun 16;11(6):e0157505. doi: 10.1371/journal.pone.0157505 (PMC4911152; doi:10.1371/journal.pone.0157505)
Supplement: S1 Table — (DOCX) [file pone.0157505.s003.docx]

S1 Table. Primer sequences and expected amplicon sizes for each locus. Primers for *mat*K, *rbc*L, and ITS2 are from the Canadian Centre for DNA Barcoding (CCDB) protocols (http://www.ccdb.ca/resources.php) and the *trn*L primers are from the 2007 study by Taberlet *et al.*

| **Locus** | **Expected Size** | **Primer Name** | **Sequence** |
| --- | --- | --- | --- |
| *mat*K | 840 bp | MatK-1RKIM-f | 5' ACCCAGTCCATCTGGAAATCTTGGTTC 3' |
|  |  | MatK-3FKIM-r | 5' CGTACAGTACTTTTGTGTTTACGAG 3' |
| *rbc*L a | 550 bp | rbcLa-F | 5' ATGTCACCACAAACAGAGACTAAAGC 3' |
|  |  | rbcLa-R | 5' GTAAAATCAAGTCCACCRCG 3' |
| ITS2 | 300-460 bp | ITS2-S2F | 5' ATGCGATACTTGGTGTGAAT 3' |
|  |  | ITS4 | 5' TCCTCCGCTTATTGATATGC 3' |
| *trn*L intron P6 loop | 10-143 bp | g | 5' GGGCAATCCTGAGCCAA 3' |
|  |  | h | 5' CCATTGAGTCTCTGCACCTATC 3' |
