# Supplementary material for: Large-Scale Monitoring of Plants through Environmental DNA Metabarcoding of Soil: Recovery, Resolution, and Annotation of Four DNA Markers
Source: PLoS One. 2016 Jun 16;11(6):e0157505. doi: 10.1371/journal.pone.0157505 (PMC4911152; doi:10.1371/journal.pone.0157505)
Supplement: S2 Table — (DOCX) [file pone.0157505.s004.docx]

S2 Table. Optimized PCR conditions used for first round amplification of each locus.

| **PCR #1** | *mat*K | *rbc*L | **ITS2** | *trn*L **P6** |
| --- | --- | --- | --- | --- |
| PCR Buffer^1^ | 1x | 1x | 1x | 1x |
| MgCl_2_^1^ | 2mM | 2mM | 2mM | 2mM |
| dNTP mix^2^ | 0.2mM | 0.2mM | 0.2mM | 0.2mM |
| Forward Primer^3^ | 0.5μM | 0.2μM | 0.2μM | 0.1μM |
| Reverse Primer^3^ | 0.5μM | 0.2μM | 0.2μM | 0.1μM |
| Platinum® Taq DNA Polymerase^1^ | 0.1U/μL | 0.1U/μL | 0.1U/μL | 0.1U/μL |
| DNA Template | 3μL | 2μL | 2μL | 2μL |
| ***Total Volume*** | ***25μL*** | ***25μL*** | ***25μL*** | ***25μL*** |

^1^ Life Technologies; Burlington, Ontario, Canada

^2^ Kapa Biosystems; Wilmington, Massachusetts, USA

^3^ IDT; Coralville, Iowa, USA
