# Supplementary material for: Large-Scale Monitoring of Plants through Environmental DNA Metabarcoding of Soil: Recovery, Resolution, and Annotation of Four DNA Markers
Source: PLoS One. 2016 Jun 16;11(6):e0157505. doi: 10.1371/journal.pone.0157505 (PMC4911152; doi:10.1371/journal.pone.0157505)
Supplement: S3 Table — (DOCX) [file pone.0157505.s005.docx]

S3 Table. Thermocycler programs used with each locus for first and second rounds of amplification.

| **Cycler Conditions** | | *mat*K | | *rbc*L | | ITS2 | | *trn*L intron P6 loop | |
| --- | --- | --- | --- | --- | --- | --- | --- | --- | --- |
| Initial | | 94°C | 5 min | 94°C | 4 min | 94°C | 5 min | 95°C | 10 min |
| Cycle | Denature | 94°C | 30 s | 94°C | 30 s | 94°C | 30 s | 95°C | 30 s |
|  | Anneal | 48°C | 20 s | 55°C | 30 s | 50°C | 30 s | 50°C | 30 s |
|  | Extend | 72°C | 50 s | 72°C | 1 min | 72°C | 45 s | -- | -- |
| Final extension | | 72°C | 5 min | 72°C | 10 min | 72°C | 10 min | -- | -- |
| Hold | | 10°C | -- | 10°C | -- | 10°C | -- | 10°C | -- |
