# Supplementary material for: Large-Scale Monitoring of Plants through Environmental DNA Metabarcoding of Soil: Recovery, Resolution, and Annotation of Four DNA Markers
Source: PLoS One. 2016 Jun 16;11(6):e0157505. doi: 10.1371/journal.pone.0157505 (PMC4911152; doi:10.1371/journal.pone.0157505)
Supplement: S5 Table — (DOCX) [file pone.0157505.s007.docx]

S5 Table. Search criteria used to build reference databases for each locus from NCBI's GenBank. Reference databases were filtered to remove sequences with more than five consecutive N’s prior to making taxonomic assignments.

| **Locus** | **Download Date** | **Search String** | **No. of Sequences** |
| --- | --- | --- | --- |
| *mat*K | 2014/09/30 | matK[title] OR "maturase K"[title] AND 0:5000[Sequence Length] NOT unverified NOT pseudogene | 84,131 |
| *rbc*L | 2014/03/17 | rbcL[gene] AND 0:5000[Sequence Length] NOT pseudogene NOT unverified | 107,555 |
| ITS2 | 2014/09/25 | (its2[title] OR internal transcribed spacer 2[title]) AND eukaryot*[organism] AND 200:2000[Sequence Length] NOT pseudogene NOT unverified | 660,667 |
| *trn*L | 2014/09/30 | ("green plants"[porgn:__txid33090]) AND 0:5000[Sequence Length] AND (trnL[title] OR "tRNA-Leu"[title]) NOT pseudogene NOT unverified | 110,088 |
