# Supplementary material for: Large-Scale Monitoring of Plants through Environmental DNA Metabarcoding of Soil: Recovery, Resolution, and Annotation of Four DNA Markers
Source: PLoS One. 2016 Jun 16;11(6):e0157505. doi: 10.1371/journal.pone.0157505 (PMC4911152; doi:10.1371/journal.pone.0157505)
Supplement: S6 Table — (DOCX) [file pone.0157505.s008.docx]

**S6 Table. Statistical test output for all analyses.**

Statistical output for DNA marker comparisons of (A) nearest neighbour distance (NND) at the species level, (B) sequence recovery with the taxonomy pipeline (i) and OTU pipeline (ii), (C) taxonomic resolution, (D) pooled soil core richness, and (E) distances to spatial medians in the principal coordinates analysis (PCoA).

**A. Nearest neighbour distance**

| **Taxonomic Level** | **Statistical Test** | **Model** | | | |
| --- | --- | --- | --- | --- | --- |
| Species | Friedman Rank Sum | NND ~ Marker + Taxa | | | |
| **Factor** | **Test Statistic** | **Degrees of Freedom** | | **p-value** | |
| Marker | Χ^2^ = 114.4925 | 3 | | <0.0001 | |
| **Factor** | **Post hoc** | **Pairwise comparison p-values** | | | |
| Marker | Wilcoxon signed rank test |  | ITS2 | matK | rbcL |
|  |  | matK | <0.0001 |  |  |
|  |  | rbcL | <0.0001 | 0.0001 |  |
|  |  | trnL | 0.0001 | 0.7827 | 0.0403 |

**B. Sequence recovery**

**i. Taxonomy pipeline**

| **Processing Level** | **Statistical Test** | | **Model** | | | | | |
| --- | --- | --- | --- | --- | --- | --- | --- | --- |
| Raw Sequences | ANOVA | | Raw Sequences ~ DNA Marker + Soil Sample | | | | | |
| **Factor** | **Df** | **SS** | **MS** | | **F value** | | **p-value** | |
| DNA Marker | 3 | 9.708 x 10^10^ | 3.236 x 10^10^ | | 2.577 | | 0.0578 | |
| Soil Sample | 34 | 2.265 x 10^11^ | 6.663 x 10^9^ | | 0.531 | | 0.9813 | |
| Residuals | 102 | 1.251 x 10^12^ | 1.256 x 10^10^ | |  | |  | |
| **Processing Level** | **Statistical Test** | | **Model** | | | | | |
| Filtered Sequences | Friedman Rank Sum | | Filtered Sequences ~ DNA Marker + Soil Sample | | | | | |
| **Factor** | **Test Statistic** | | **Degrees of Freedom** | | | **p-value** | | |
| DNA Marker | Χ^2^ = 46.0286 | | 3 | | | <0.0001 | | |
| **Factor** | **Post hoc** | | **Pairwise comparison p-values** | | | | | |
| DNA Marker | Wilcoxon signed rank test | |  | ITS2 | | matK | | rbcL |
|  |  |  | matK | <0.0001 | |  | |  |
|  |  |  | rbcL | 0.0006 | | 0.0550 | |  |
|  |  |  | trnL | 0.1488 | | <0.0001 | | 0.0001 |
| **Processing Level** | **Statistical Test** | | **Model** | | | | | |
| BLAST Hits | Friedman Rank Sum | | Sequences with BLAST hit ~ DNA Marker + Soil Sample | | | | | |
| **Factor** | **Test Statistic** | | **Degrees of Freedom** | | | **p-value** | | |
| DNA Marker | Χ^2^ = 171.0571 | | 3 | | | <0.0001 | | |
| **Factor** | **Post hoc** | | **Pairwise comparison p-values** | | | | | |
| DNA Marker | Wilcoxon signed rank test | |  | ITS2 | | matK | | rbcL |
|  |  |  | matK | <0.0001 | |  | |  |
|  |  |  | rbcL | 0.3423 | | <0.0001 | |  |
|  |  |  | trnL | <0.0001 | | <0.0001 | | 0.0005 |
| **Processing Level** | **Statistical Test** | | **Model** | | | | | |
| Assigned Orders | Friedman Rank Sum | | Sequences Assigned to Order ~ DNA Marker + Soil Sample | | | | | |
| **Factor** | **Test Statistic** | | **Degrees of Freedom** | | | **p-value** | | |
| DNA Marker | Χ^2^ = 45.96 | | 3 | | | <0.0001 | | |
| **Factor** | **Post hoc** | | **Pairwise comparison p-values** | | | | | |
| DNA Marker | Wilcoxon signed rank test | |  | ITS2 | | matK | | rbcL |
|  |  |  | matK | <0.0001 | |  | |  |
|  |  |  | rbcL | 0.891 | | <0.0001 | |  |
|  |  |  | trnL | 0.069 | | <0.0001 | | 0.222 |
| **Processing Level** | **Statistical Test** | | **Model** | | | | | |
| Assigned Vascular | Friedman Rank Sum | | Sequences Assigned Vascular ~ DNA Marker + Soil Sample | | | | | |
| **Factor** | **Test Statistic** | | **Degrees of Freedom** | | | **p-value** | | |
| DNA Marker | Χ^2^ = 24.5657 | | 3 | | | <0.0001 | | |
| **Factor** | **Post hoc** | | **Pairwise comparison p-values** | | | | | |
| DNA Marker | Wilcoxon signed rank test | |  | ITS2 | | matK | | rbcL |
|  |  |  | matK | 0.0003 | |  | |  |
|  |  |  | rbcL | 0.0940 | | <0.0001 | |  |
|  |  |  | trnL | 0.9549 | | <0.0001 | | 0.2619 |

**ii. OTU pipeline**

| **Processing Level** | **Statistical Test** | **Model** | | | |
| --- | --- | --- | --- | --- | --- |
| OTU Sequences | Friedman Rank Sum | Sequences Assigned OTUs ~ DNA Marker + Soil Sample | | | |
| **Factor** | **Test Statistic** | **Degrees of Freedom** | | **p-value** | |
| DNA Marker | Χ^2^ = 74.0057 | 3 | | <0.0001 | |
| **Factor** | **Post hoc** | **Pairwise comparison p-values** | | | |
| DNA Marker | Wilcoxon signed rank test |  | ITS2 | matK | rbcL |
|  |  | matK | <0.0001 |  |  |
|  |  | rbcL | 0.0002 | <0.0001 |  |
|  |  | trnL | <0.0001 | <0.0001 | <0.0001 |
| **Processing Level** | **Statistical Test** | **Model** | | | |
| Vascular Plant OTUs | Friedman Rank Sum | Sequences Assigned to Vascular Plant OTUs ~ DNA Marker + Soil Sample | | | |
| **Factor** | **Test Statistic** | **Degrees of Freedom** | | **p-value** | |
| DNA Marker | Χ^2^ = 61.5257 | 3 | | <0.0001 | |
| **Factor** | **Post hoc** | **Pairwise comparison p-values** | | | |
| DNA Marker | Wilcoxon signed rank test |  | ITS2 | matK | rbcL |
|  |  | matK | 0.0022 |  |  |
|  |  | rbcL | 0.2516 | <0.0001 |  |
|  |  | trnL | <0.0001 | <0.0001 | <0.0001 |

**C. Taxonomic resolution**

| **Taxonomic Level** | **Statistical Test** | **Model** | | | |
| --- | --- | --- | --- | --- | --- |
| Family:Order | Friedman Rank Sum | (1-(Family/Order Sequences))~DNA Marker + Soil Sample | | | |
| **Factor** | **Test Statistic** | **Degrees of Freedom** | | **p-value** | |
| DNA Marker | Χ^2^ = 69.7909 | 3 | | <0.0001 | |
| **Factor** | **Post hoc** | **Pairwise comparison p-values** | | | |
| DNA Marker | Wilcoxon signed rank test |  | ITS2 | matK | rbcL |
|  |  | matK | <0.0001 |  |  |
|  |  | rbcL | <0.0001 | 0.068 |  |
|  |  | trnL | <0.0001 | <0.0001 | 0.581 |
| **Taxonomic Level** | **Statistical Test** | **Model** | | | |
| Genus:Order | Friedman Rank Sum | (1-(Genus/Order Sequences))~DNA Marker + Soil Sample | | | |
| **Factor** | **Test Statistic** | **Degrees of Freedom** | | **p-value** | |
| DNA Marker | Χ^2^ = 84.5415 | 3 | | <0.0001 | |
| **Factor** | **Post hoc** | **Pairwise comparison p-values** | | | |
| DNA Marker | Wilcoxon signed rank test |  | ITS2 | matK | rbcL |
|  |  | matK | <0.0001 |  |  |
|  |  | rbcL | <0.0001 | 0.32 |  |
|  |  | trnL | <0.0001 | <0.0001 | <0.0001 |
| **Taxonomic Level** | **Statistical Test** | **Model** | | | |
| Species:Order | Friedman Rank Sum | (1-(Species/Order Sequences))~DNA Marker + Soil Sample | | | |
| **Factor** | **Test Statistic** | **Degrees of Freedom** | | **p-value** | |
| DNA Marker | Χ^2^ = 20.6229 | 3 | | <0.0001 | |
| **Factor** | **Post hoc** | **Pairwise comparison p-values** | | | |
| DNA Marker | Wilcoxon signed rank test |  | ITS2 | matK | rbcL |
|  |  | matK | 0.1211 |  |  |
|  |  | rbcL | 0.0001 | <0.0001 |  |
|  |  | trnL | 0.1211 | 0.3260 | 0.0296 |

**D. Pooled soil core richness**

| **Taxonomic Level** | **Statistical Test** | | **Model** | | | | | |
| --- | --- | --- | --- | --- | --- | --- | --- | --- |
| Order | ANOVA | | Richness~ DNA Marker + Sampling Instance | | | | | |
| **Factor** | **Df** | **SS** | **MS** | | **F value** | | **p-value** | |
| DNA Marker | 3 | 214.9 | 71.64 | | 18.035 | | <0.0001 | |
| Sampling Instance | 11 | 221.9 | 20.17 | | 5.079 | | 0.0001 | |
| Residuals | 33 | 131.1 | 3.97 | |  | |  | |
| **Factor** | **Post hoc** | | **Pairwise comparison p-values** | | | | | |
| DNA Marker | Tukey’s HSD | |  | ITS2 | | matK | | rbcL |
|  |  |  | matK | 0.7366 | |  | |  |
|  |  |  | rbcL | 0.0043 | | 0.0002 | |  |
|  |  |  | trnL | <0.0001 | | <0.0001 | | 0.3717 |
| **Taxonomic Level** | **Statistical Test** | | **Model** | | | | | |
| Family | ANOVA | | Richness~ DNA Marker + Sampling Instance | | | | | |
| **Factor** | **Df** | **SS** | **MS** | | **F value** | | **p-value** | |
| DNA Marker | 3 | 364.4 | 121.47 | | 15.606 | | <0.0001 | |
| Sampling Instance | 11 | 342.1 | 31.10 | | 3.995 | | 0.0010 | |
| Residuals | 33 | 256.9 | 7.78 | |  | |  | |
| **Factor** | **Post hoc** | | **Pairwise comparison p-values** | | | | | |
| DNA Marker | Tukey’s HSD | |  | ITS2 | | matK | | rbcL |
|  |  |  | matK | 0.9358 | |  | |  |
|  |  |  | rbcL | 0.0038 | | <0.0001 | |  |
|  |  |  | trnL | <0.0001 | | <0.0001 | | 0.5142 |
| **Taxonomic Level** | **Statistical Test** | | **Model** | | | | | |
| Genus | ANOVA | | Richness~ DNA Marker + Sampling Instance | | | | | |
| **Factor** | **Df** | **SS** | **MS** | | **F value** | | **p-value** | |
| DNA Marker | 3 | 743.2 | 247.72 | | 10.285 | | <0.0001 | |
| Sampling Instance | 11 | 837.7 | 76.15 | | 3.162 | | 0.0051 | |
| Residuals | 33 | 794.8 | 24.09 | |  | |  | |
| **Factor** | **Post hoc** | | **Pairwise comparison p-values** | | | | | |
| DNA Marker | Tukey’s HSD | |  | ITS2 | | matK | | rbcL |
|  |  |  | matK | 0.9993 | |  | |  |
|  |  |  | rbcL | 0.0006 | | 0.0008 | |  |
|  |  |  | trnL | 0.0080 | | 0.0111 | | 0.7746 |
| **Taxonomic Level** | **Statistical Test** | | **Model** | | | | | |
| OTU | ANOVA | | Log10(Richness)~DNA Marker + Sampling Instance | | | | | |
| **Factor** | **Df** | **SS** | **MS** | | **F value** | | **p-value** | |
| DNA Marker | 3 | 5.735 | 1.9118 | | 19.658 | | <0.0001 | |
| Sampling Instance | 11 | 0.607 | 0.0552 | | 0.568 | | 0.841 | |
| Residuals | 33 | 3.209 | 0.0973 | |  | |  | |
| **Factor** | **Post hoc** | | **Pairwise comparison p-values** | | | | | |
| DNA Marker | Tukey’s HSD | |  | ITS2 | | matK | | rbcL |
|  |  |  | matK | 0.9939 | |  | |  |
|  |  |  | rbcL | 0.0026 | | 0.0013 | |  |
|  |  |  | trnL | <0.0001 | | <0.0001 | | 0.1109 |

**E.** **PCoA distances**

| **Taxonomic Level** | **Statistical Test** | | **Model** | | | | | |
| --- | --- | --- | --- | --- | --- | --- | --- | --- |
| Order | ANOVA | | Distance~DNA Marker + Sampling Instance | | | | | |
| **Factor** | **Df** | **SS** | **MS** | | **F value** | | **p-value** | |
| DNA Marker | 3 | 0.0118 | 0.00394 | | 0.184 | | 0.906 | |
| Sampling Instance | 11 | 0.2068 | 0.01880 | | 0.878 | | 0.569 | |
| Residuals | 33 | 0.7063 | 0.02140 | |  | |  | |
| **Taxonomic Level** | **Statistical Test** | | **Model** | | | | | |
| Family | ANOVA | | Distance~DNA Marker + Sampling Instance | | | | | |
| **Factor** | **Df** | **SS** | **MS** | | **F value** | | **p-value** | |
| DNA Marker | 3 | 0.1059 | 0.03531 | | 2.375 | | 0.0879 | |
| Sampling Instance | 11 | 0.1857 | 0.01688 | | 1.135 | | 0.3669 | |
| Residuals | 33 | 0.4907 | 0.01487 | |  | |  | |
| **Taxonomic Level** | **Statistical Test** | | **Model** | | | | | |
| Genus | ANOVA | | Distance~DNA Marker + Sampling Instance | | | | | |
| **Factor** | **Df** | **SS** | **MS** | | **F value** | | **p-value** | |
| DNA Marker | 3 | 0.3984 | 0.13280 | | 15.280 | | <0.0001 | |
| Sampling Instance | 11 | 0.0960 | 0.00873 | | 1.005 | | 0.463 | |
| Residuals | 33 | 0.2868 | 0.00869 | |  | |  | |
| **Factor** | **Post hoc** | | **Pairwise comparison p-values** | | | | | |
| DNA Marker | Tukey’s HSD | |  | ITS2 | | matK | | rbcL |
|  |  |  | matK | 0.7498 | |  | |  |
|  |  |  | rbcL | 0.4516 | | 0.0036 | |  |
|  |  |  | trnL | 0.0001 | | <0.0001 | | 0.1049 |
